# Fluoroscopic evaluation of diaphragmatic motion reduction with a respiratory gated radiotherapy system

**DOI:** 10.1120/jacmp.v2i4.2596

**Published:** 2001-09-01

**Authors:** Gikas S. Mageras, Ellen Yorke, Kenneth Rosenzweig, Louise Braban, Eric Keatley, Eric Ford, Steven A. Leibel, C. Clifton Ling

**Affiliations:** ^1^ Department of Medical Physics Memorial Sloan Kettering Cancer Center 1275 York Avenue New York New York 10021; ^2^ Department of Radiation Oncology Memorial Sloan Kettering Cancer Center 1275 York Avenue New York New York 10021

**Keywords:** respiration, gating, radiotherapy

## Abstract

We report on initial patient studies to evaluate the performance of a commercial respiratory gating radiotherapy system. The system uses a breathing monitor, consisting of a video camera and passive infrared reflective markers placed on the patient's thorax, to synchronize radiation from a linear accelerator with the patient's breathing cycle. Six patients receiving treatment for lung cancer participated in a study of system characteristics during treatment simulation with fluoroscopy. Breathing synchronized fluoroscopy was performed initially without instruction, followed by fluoroscopy with recorded verbal instruction (i.e., when to inhale and exhale) with the tempo matched to the patient's normal breathing period. Patients tended to inhale more consistently when given instruction, as assessed by an external marker movement. This resulted in smaller variation in expiration and inspiration marker positions relative to total excursion, thereby permitting more precise gating tolerances at those parts of the breathing cycle. Breathing instruction also reduced the fraction of session times having irregular breathing as measured by the system software, thereby potentially increasing the accelerator duty factor and decreasing treatment times. Fluoroscopy studies showed external monitor movement to correlate well with that of the diaphragm in four patients, whereas time delays of up to 0.7 s in diaphragm movement were observed in two patients with impaired lung function. From fluoroscopic observations, average patient diaphragm excursion was reduced from 1.4 cm (range 0.7–2.1 cm) without gating and without breathing instruction, to 0.3 cm (range 0.2–0.5 cm) with instruction and with gating tolerances set for treatment at expiration for 25% of the breathing cycle. Patients expressed no difficulty with following instruction for the duration of a session. We conclude that the external monitor accurately predicts internal respiratory motion in most cases; however, it may be important to check with fluoroscopy for possible time delays in patients with impaired lung function. Furthermore, we observe that verbal instruction can improve breathing regularity, thus improving the performance of gated treatments with this system.

PACS number(s): 87.53.–j, 87.62.+n

## INTRODUCTION

Respiratory motion in the thorax and abdomen is an important limiting factor in high‐precision radiation therapy. Movement of 1–3 cm during quiet breathing has been reported in the lung, liver, and kidney.^1–^, ^8^ A correspondingly large planning target volume is therefore required to avoid marginal misses. Breathing motion can also produce inaccuracies in computed tomography (CT) based treatment plans. Sizeable errors in organ volume, position, and shape can occur, which can significantly affect the calculation of dose‐volume histograms.^9,^, ^10^ Intrafractional organ motion can affect intensity‐modulated radiotherapy (IMRT) delivered dynamically with multileaf collimators.^11,^, ^12^ In this type of treatment, an intensity‐modulated field is composed essentially of many small fields that are delivered temporally; thus, the dose distributions actually received by the moving target and nontarget organs can be different from the ones that were planned.

Two different interventional strategies have evolved to reduce the effect of respiratory motion in radiation treatments: controlled patient breathing, and respiration gating of the accelerator while the patient breathes normally. In the former approach, breathing is altered either voluntarily by instructing the patient,^5,^, ^13–^, ^16^ or assisted by means of an occlusion valve.^17,^, ^19^ In the latter approach, a device monitors patient breathing and allows delivery of radiation only during certain time intervals, synchronous with the patient's respiratory cycle.^1,^, ^20–^, ^28^

We have previously reported on the use of a deep inspiration breath‐hold (DIBH) technique in conformal radiation treatments of nonsmall cell lung carcinoma.^5,^, ^14,^, ^15^ Using spirometry as a monitor of lung volume, the patient is coached through a modified slow vital capacity maneuver to achieve a reproducible inspiration level. The maneuver consists of a slow deep inspiration, slow deep expiration, then another slow deep inspiration to maximal inspiratory level and breath‐hold. The potential advantages of the technique are twofold: the breath‐hold immobilizes the tumor, and deep inspiration reduces the normal lung density relative to the tumor, thus reducing the mass of the normal lung receiving a high dose. However, roughly one‐third to one‐half of eligible patients could not perform the DIBH technique satisfactorily, and average session times for simulation and treatment of the initial patients were nearly double that for free‐breathing treatments. Moreover, deep inspiration may not be an advantage in other disease sites subject to respiratory motion, such as liver. In contrast, respiration gating with the patient breathing normally is potentially less demanding and thus more generally applicable. For these reasons, we have investigated respiration gating as an alternative interventional strategy, and report here on initial patient studies at our center of a commercial respiration gating radiotherapy system.

## METHODS AND MATERIALS

The gated radiotherapy system used for these studies (Real‐Time Position Management Respiratory Gating System, Varian Medical Systems, Palo Alto, CA) permits breathing‐synchronized fluoroscopy on a treatment simulator, as well as gated treatment on a linear accelerator^24,^, ^29^ The system consists of a desktop computer equipped with real‐time digital video acquisition and display, gating software and user interface, a charge‐coupled‐device (CCD) video camera with an attached infrared illuminator, and a linear accelerator gating interface for beam on‐off control. To monitor respiration, a lightweight block containing two passive reflective markers is placed on the patient's chest or abdomen. Infrared light from an illuminator is reflected from the markers and detected by the CCD camera. The upper marker serves to track respiratory movement, while the lower marker, separated from the upper one by 3 cm, serves to calibrate the system. The video signal from the camera is processed by a software application running on the desktop computer. At the start of any session, whether simulation or treatment, the operator places the system into a so‐called tracking mode for a few breathing cycles, to allow the system to determine the minimum and maximum vertical position of the upper marker. These values establish the scale of the marker motion for the purposes of display and for setting thresholds, described below. In addition, a periodicity filter algorithm checks that the breathing wave form (i.e., the marker position versus time) is regular and periodic. Once the operator has verified that the minimum and maximum positions are stable and breathing is regular, the operator places the system into a record mode, during which the breathing wave form is recorded and displayed. User‐adjustable threshold levels are superimposed as two horizontal lines on the wave form, and are calculated relative to the minimum and maximum marker position measured during the tracking mode. During treatment, the beam is delivered only when the breathing wave form is between the upper and lower gating threshold lines. In addition, the periodicity filter immediately disables the treatment beam in the event of an irregular breathing wave form, such as patient movement, coughing or sighing, and re‐enables the beam after establishing that breathing is again regular. An additional display at the bottom of the computer screen indicates when the beam is enabled.

**Figure 1:**
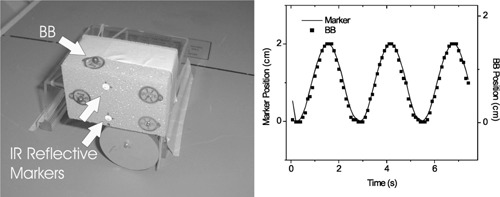
(a) Mechanical motion phantom and reflective marker block. (b) Comparison of the marker position wave form vs time from the gating system camera, with the BB position observed from fluoroscopic images.

On a treatment simulator, the gating system allows recording and playback of fluoroscopy images from the image intensifier, synchronized with the external breathing motion wave form. The fluoroscopic images are recorded at a rate of 10 frames/s. The breathing wave form is sampled 30 times/s and recorded in a data file, along with the corresponding fluoroscopic image frame number and status of the periodicity filter (i.e., regular or irregular breathing) for each sample. The user specifies a treatment point, or gate, in the breathing cycle by adjusting the threshold levels with respect to the breathing motion wave form. Only those fluoroscopy frames occurring during the gate intervals are played back. The operator then examines anatomic motion in the playback to evaluate and optimize the choice of gating thresholds. A test performed with a mechanical motion phantom shows excellent synchronization between the wave form of the camera‐detected marker position, and the position of a radio‐opaque marker, or BB, detected in the fluoroscopic movie (Fig. 1).

Six patients treated for lung cancer participated in a fluoroscopic study to evaluate the gating system. All patients underwent fluoroscopy of approximately one‐minute duration, while breathing normally during the simulation session. Five patients (Patients 2–6) were then asked to follow simple verbal instruction, (i.e., “breathe in, breathe out”), that was recorded at a tempo slightly slower (by approximately 1 second) than the patient's normal breathing rate. The verbal instruction was recorded with commercial software (Cool Edit, Symantiac Software Corporation, Phoenix, AZ), which allowed sound track editing and could be played back in a loop mode. The patients were trained for approximately 2–3 min with instruction, to ensure that they were comfortable with the breathing tempo, and to make any adjustments if necessary. A second fluoroscopy was then recorded with breathing instruction.

The relationship between diaphragm and marker positions versus time was examined in the fluoroscopy movies, to assess the degree of correlation between external marker and internal anatomic motion. Measurement of the diaphragm apex position in fluoroscopy movies was accomplished by means of a computer‐automated program developed in‐house for this purpose.^30^ The diaphragm position versus time was then correlated with the breathing wave form by means of the system‐recorded data file described above. In order to quantify the amount of diaphragm movement, the measurements of diaphragm position were made at 100‐ms intervals in the movie, sorted in order of increasing position, and the 10th and 90th percentiles chosen, which represented the diaphragm excursion. This calculation is less sensitive to outliers (e.g., a single deep breath) than by taking the extremes of diaphragm position in a session. This calculation was repeated for all the fluoroscopy data in a session, and for data within a given gate interval based on the respiration wave form, for comparing diaphragm movement without and with gating, respectively.

## RESULTS

In baseline studies with volunteers from our staff, we determined that positioning the marker block midway between the xyphoid tip and umbilicus yielded the largest amplitude in marker motion of typically 1–2 cm. With patients it was sometimes necessary to adjust marker location until sufficient amplitude (at least 5 mm) was observed. The chosen location was then marked on the patient in order to reposition it for subsequent treatment sessions.

**Figure 2:**
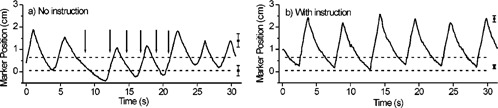
Example breathing wave forms from different sessions of the same patient, (a) without and (b) with breathing instruction. Horizontal lines indicate gate threshold levels, set for intended treatment at end expiration. Arrows in (a) indicate locations in the breathing cycle that result in unintended beam enable signals. At the right of each figure, square symbols and error bars indicate the mean (over the session) and one standard deviation in marker peak position, respectively, at end inspiration and end expiration.

Soon after patient studies were initiated, we observed that changes in patient breathing affected the performance of the respiratory gating system. Figure 2(a) shows the breathing wave form of Patient 1 without breathing instruction. The thresholds were set for treatment at end expiration at the beginning of the session; however, the breathing wave form was irregular during the session resulting in beam enable signals at unintended points in the breathing cycle (arrows). Figure 2(b) shows a breathing wave form for the same patient, but with breathing instruction. The more regular breathing pattern with instruction is evident, with smaller variation in the positions of the maxima (end inspiration) and minima (end expiration) from one cycle to the next (error bars at right), as well as smaller variation in the breathing period. In addition, patients tended to inhale more deeply when given breathing instruction, as evidenced by the larger excursion in marker position.

**Figure 3:**
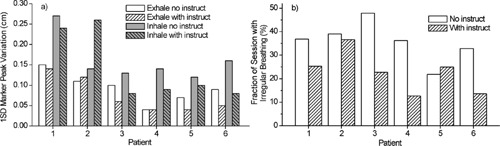
(a) Comparison of one‐standard‐deviation variation in external marker position at end expiration and inspiration for each patient, for sessions with and without breathing instruction. (b) Fraction of session time in which irregular breathing occurred as measured by the system periodicity algorithm.

External marker motion data for the six patients are summarized in Fig. 3. The one‐standard‐deviation (1SD) variation in marker peak position at end expiration (inspiration) is calculated for each session, using the minima (maxima) in the breathing wave form, then averaged over sessions. The variation at end expiration is reduced with instruction in four out of six patients, whereas at end inspiration it is reduced in five out of six patients [Fig. 3(a)]. In addition, the fraction of session time having irregular breathing as measured by the periodicity filter (i.e., the fraction of wave form samples with irregular breathing status, obtained from the system‐recorded data file described in the Methods section) is reduced in five out of six patients with instruction [Fig. 3(b)]. For the six patients, the fraction of total time during gated operation that the patient is breathing irregularly (thus inhibiting treatment) is decreased from an average of 36% (range 22–48%) without instruction, to 23% with instruction (range 13–37%).

**Figure 4:**
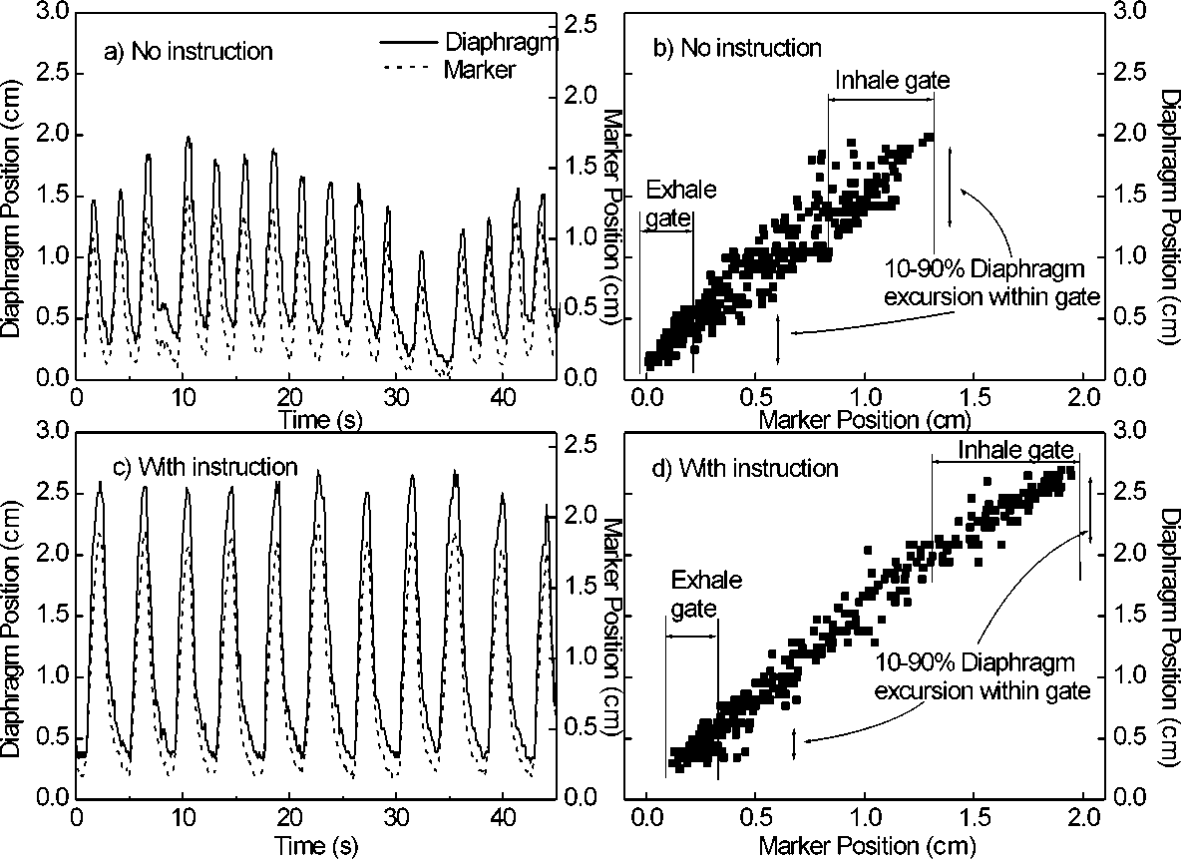
(a) Comparison of breathing wave form (marker position) and diaphragm position vs time of Patient 4, for a session with no breathing instruction. (b) Scatter plot of diaphragm position vs marker position for the same session. Vertical lines indicate thresholds for intended treatment at expiration (“exhale gate”) for 25% of the breathing cycle, and for treatment at inspiration (“inhale gate”). Vertical double‐arrow lines indicate the 10–90% diaphragm excursion occurring within the gate intervals. (c) and (d): same as (a) and (b), but for a session with breathing instruction.

From the recorded fluoroscopic and breathing wave form data, we examined how well the external marker predicts internal motion, and to what extent this is influenced by breathing instruction. Figure 4(a) compares the marker position versus time with the observed diaphragm position of a patient without breathing instruction, while Fig. 4(b) shows the same data as a scatter plot of diaphragm versus marker position. The data show a high degree of correlation between external monitor and internal anatomy, even in the presence of some irregular breathing (linear correlation coefficient R=0.95). Breathing instruction serves to increase the degree of correlation in this patient [R=0.99, Figs. 4(c) and 4(d)].

**Figure 5:**
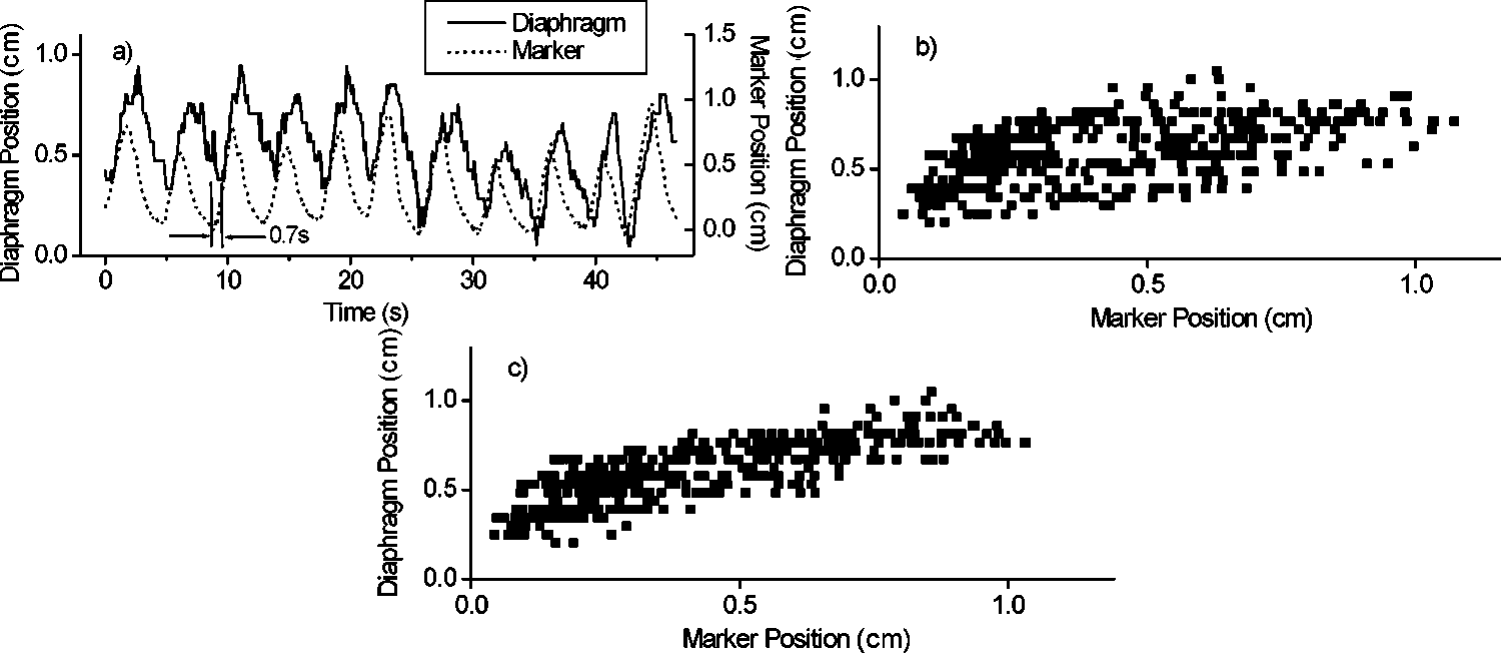
(a) Comparison of breathing wave form and diaphragm position vs time in an instructed fluoroscopy session of Patient 5. Note the phase delay of 0.7 s in diaphragm position at end expiration, relative to the breathing wave form (arrows in lower left). (b) Scatter plot of diaphragm vs marker position, illustrating the reduced correlation (compare Fig. 4). (c) Scatter plot after shifting diaphragm position vs time in (a) forward by 0.7 s, resulting in improved correlation.

A similar high degree of correlation is observed in four out of six patients (mean R=0.66, range 0.95 to 0.99). Patients 2 and 5, however, show less correlation (R=0.66 and 0.53, respectively), owing to phase delays in the diaphragm movement of the diseased lung (0.5 s and 0.7 s, respectively), relative to the external marker movement (Fig. 5 shows data for Patient 5). When the diaphragm position versus time is advanced by the observed phase delay, the correlation improves to R=0.79 and 0.81, respectively [Fig. 5(c)].

**Figure 6:**
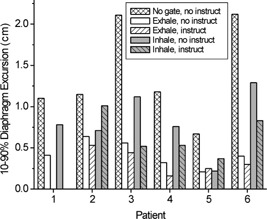
Comparison of 10–90% diaphragm excursion measured with fluoroscopy under five different conditions: no gating and no instruction, gate thresholds set at expiration (see Fig. 4) without and with breathing instruction, thresholds set at inspiration without and with instruction.

By setting thresholds on marker position for intended treatment at end expiration and for 25% of the breathing cycle [“exhale gate” in Figs. 4(b) and 4(d)], we determine the corresponding 10–90% diaphragm excursion within the gate interval (double‐arrow lines) and compare fluoroscopic sessions with and without instruction (see the Methods section for the calculation of 10–90% excursion). Similarly, we make this comparison for thresholds set at end inspiration (“inhale gate”). The results for all patients are shown in Fig. 6. In the five patients for whom fluoroscopic data with and without breathing instruction are available (Patients 2 through 6), instruction had a modest effect in reducing diaphragm excursion at expiration, i.e., 0.15 cm or less (compare white open and white hatched bars). At inspiration, instruction had a more pronounced effect, with three out of five patients showing a reduction in diaphragm excursion between 0.2 and 0.5 cm (compare gray open and gray hatched bars). The two patients (Patients 2 and 5) in whom diaphragm excursion was larger were those with reduced correlation between respiration wave form and the diaphragm of the diseased lung (Fig. 5); in addition, Patient 2 exhibited irregular breathing despite instruction.

Figure 6 also shows the degree to which the combination of gating and breathing instruction reduces diaphragm movement in the fluoroscopic studies, relative to no gating and no instruction (white cross‐hatched bars). A factor of 2 to 5 reduction in diaphragm excursion is achievable with gating thresholds set at end expiration for 25% of the breathing cycle (white hatched bars), and a factor of 1.2 to 4 at end inspiration (gray hatched bars). Patient averaged diaphragm excursion with neither gating nor instruction is 1.4 cm (range 0.7–2.1 cm), which is reduced with breathing instruction to an average 0.3 cm (range 0.2–0.5 cm) with gating at expiration, and 0.7 cm (range 0.4–1.0 cm) with gating at end inspiration.

## DISCUSSION

The fluoroscopic studies show that in most patients, movement of an external respiratory monitor, placed approximately midway between the xyphoid tip and umbilicus, correlates well with diaphragmatic motion. Since the abdominal contents are largely incompressible, the movement of the diaphragm inferiorly during inspiration usually leads to an outward displacement of the abdominal wall.^31^ It is worth noting that in a study of the relative contributions of rib cage and abdominal movement to lung volume in healthy men and women during quiet breathing, subjects exhibited predominantly abdominal breathing when in the supine position^32^ For establishing an appropriate point in the respiratory cycle for treatment, however, it may be important to evaluate the agreement between the external monitor and the internal anatomy under fluoroscopy. We note that in two patients (2 and 5) with impaired function in the diseased lung, diaphragm motion showed a clearly observable phase delay with respect to the breathing wave form (Fig. 5). In such cases, a corresponding delay in the beam gate would yield some further reduction organ motion during treatment. For example, if we simulate such a delay in the gate position at end expiration by advancing the diaphragm versus time signal for Patients 2 and 5, the diaphragm excursion during the gate interval is reduced 15% and 17%, respectively. A more recent system software release provides the option of gating on the phase of the breathing wave form rather than amplitude, which allows more flexibility in positioning the gate interval. Briefly, once the periodicity filter algorithm has established that the breathing wave form is periodic, it calculates a period for the respiratory cycle (typically 3 to 6 seconds) and assigns a phase to each point in the wave form, with the zero degree point corresponding to the wave form maximum. During the simulation session, the operator adjusts separately the phase for the start (beam enable) and end (beam disable) of the gate, and views the resultant organ motion during the gate interval in the fluoroscopy playback. Since the system currently does not provide software tools of the type used in this study to determine phase delays in organ motion, adjustment of the gate position to account for such delays must be done on a trial‐and‐error basis. However, based on the examples given above it may be sufficient to position the gate within 0.5 s of the true optimal position to adequately account for phase delays. We also note that in Patients 2 and 5, the diaphragm‐marker correlation was less than in the other patients even after applying a constant phase delay correction. This may possibly be due to variability in the phase delay over the respiratory cycle, although this was not examined in this study.

The choice of gate thresholds with this system involves a trade‐off between the amount of residual organ movement during the beam‐on intervals and the longer treatment time. This study indicates that gated treatment for 25% of the breathing cycle at expiration can achieve an accuracy of 3–5 mm in diaphragm position, with somewhat less accuracy (average 7 mm) achievable at inspiration. Gated treatment at end inspiration may be of benefit in treatment of lung cancer. A study by Paoli *et al.*, comparing free‐breathing and breath‐hold treatment plans at end inspiration suggests that increased lung inflation in the latter may reduce the probability of lung toxicity.^33^

Our initial findings in six patients indicate that verbal breathing instruction helps in the majority of patients to improve regularity in breathing, and hence improves the performance of the respiratory gated radiotherapy system studied here. First, regularity in the external marker position at expiration or inspiration is improved over a treatment session, with the improvement being more pronounced at inspiration. This reduces the likelihood of beam delivery at unintended points in the breathing cycle. Because of the position‐sensitive nature of the external monitor, when a change in the shape of the breathing wave form occurs, one cannot readily distinguish whether it is due to patient movement or to true changes in inspiration levels. Nevertheless, such a drift of the wave form with respect to the amplitude‐based thresholds can result in dose delivery occurring at points where breathing motion is largest, i.e., between end inspiration and expiration such as illustrated in Fig. 2(a).

A second benefit of breathing instruction is to substantially decrease the fraction of session time having irregular breathing as measured by the system's periodicity filter, thereby increasing the accelerator duty factor and decreasing the treatment times during gated operation. In some of the initial treatment sessions without instruction, intervals of inhibited beam delivery from irregular breathing sometimes extended over several respiratory cycles, leading to treatment interruption from under‐dose interlocks. The system does allow amplitude‐based gated operation with the periodicity filter disabled; however, that leads to the potential risk that dose delivery may not be disabled during intervals of patient cough or sudden movement. Phase‐based gating may be more robust against breathing wave form drift as discussed in the previous paragraph. However, phase‐based gating requires the periodicity filter to establish that breathing is regular and to determine breathing period, which in turn underscores the importance of achieving a consistent and regular patient breathing. Third, the data presented here suggest that breathing instruction may improve organ position accuracy in gated treatments, particularly at inspiration, as inferred from diaphragm position in fluoroscopy measurements. Although the tendency towards deeper breathing with instruction may increase diaphragm excursion, the more regular breathing wave form allows tighter gate thresholds to be set. However, patient cooperation and lung function are important factors that can affect the actual benefit of instruction, with respect to reduced internal motion. In addition, although this study indicates that reproducibility within a session is improved with verbal instruction, larger variations between sessions may occur. In such situations, visual feedback may be helpful to regulate the degree of inspiration, in which the patient sees the current respiration wave form compared to a reference one (e.g., from simulation). Such a capability will be available in a future release of the system software.

In this study we have examined the diaphragm as a measure of internal respiratory motion because the lung‐diaphragm boundary is readily detectable in fluoroscopic and portal images using automated methods and thus is a logical first step in evaluating respiratory gated treatment. More direct measurement of tumor motion is essential for assigning appropriate margins and determining optimal treatment points in the breathing cycle, however. Lung tumor motion may be influenced by costal as well as diaphragmatic breathing,^15^ or by cardiac motion, depending on tumor location within the lung. Lung elasticity and resistance to airflow can introduce phase delays in volume change and hence in tumor movement relative to intrathoracic pressure exerted by the intracostal muscles and diaphragm, the magnitude of which will depend on lung condition^34^ Terahara *et al.* have reported phase delays of 0.28 second in tumor movement during the expiration phase, relative to respiration wave forms measured with a position sensitive monitor placed on patients.^35^

The patients in this study had no difficulty in following the breathing instruction for the duration of a fluoroscopy or treatment session. Since patients tended to breathe more deeply when given instruction, a tempo slightly slower than patient's natural breathing rate was more comfortable for them to follow.

## ACKNOWLEDGMENTS

We thank Dr. Ernesto Fontenla, Dr. Agung Hertanto, and Dr. Hassan Mostafavi (Varian) for their assistance with some of the gated radiotherapy measurements. We also thank Varian Medical Systems for providing on loan the gating equipment used in this study. This work was supported in part by National Cancer Institute Grant No. 5‐PO1‐CA59017, U.S. National Institutes of Health.
